# Health-related quality of life in caregivers of community-dwelling individuals with disabilities or chronic conditions. A gender-differentiated analysis in a cross-sectional study

**DOI:** 10.1186/s12912-022-00845-x

**Published:** 2022-03-29

**Authors:** Milagros Rico-Blázquez, Víctor Quesada-Cubo, Elena Polentinos-Castro, Raquel Sánchez-Ruano, M Ángeles Rayo-Gómez, Isabel del Cura-González, CuidaCare Group, Milagros Rico-Blázquez, Rosario Almena-Martín, Ángeles Almodovar-López, Julia Alonso-Arcas, Elvira Álvarez-Navarro, Henar Álvarez-Santos, Begoña Andrés-Alonso, Virginia Antolín-Díaz, Mercedes Araujo-Calvo, Encarnación Ayuso-Gil, Cynthia A. Barbero-Macías, Inés Bermejo-Mayoral, Ana Berninches-Heredero, Lourdes Botanes-Peñafiel, Lorena Cámara-González, Isabel Careaga-González, Sergio de-Casas-Albendea, Carmen Castilla-Álvarez, Belén Castro-Sánchez, Noelia Castro-Torrado, María J. Clemente-del-Castillo, Pilar Dávila-Moriña, Juana Díaz-de-Espada-León, Analía Domínguez-González, Lorena Domínguez-Pérez, Dolores Domínguez-Puebla, Mónica Escribano-Zaera, Luisa Escudero-Muñoz, Raquel Fernández-Arnaldo, Teresa Fernández-del-Campo-Coca, Mercedes Fernández-Ortega, Rafaela Fernández-Rodríguez, Virginia García-Campo, Isabel García-del-Río, María J. García-Garrudo, Elena García-Gómez-de-Cardiñanos, Juan García-Ruíz, Petra García-San, Jorge Geanini-Torres, Rosa Gómez-Quevedo, Eva Gómez-Robledo, Carmen Gómez-Pesquera, Begoña González-Fernández, Aranzazu González-Valls, Natalie Harris-de-la-Vega, Susana Herrero-Yusta, Sonia de-la-Iglesia-Moreno, Silvia Jiménez-Maillo, Luisa Juárez-Zapatero, Raquel Juez-Pimienta, Francisca Lara-Bueno, Pilar Lasala-Raso, Lucía Letón-Gutiérrez, Margarita Leza-Leza, Raquel López-del-Cid, Laura López-Kölmer, Paz Lozano-Fernández, Elisa López-Serrano, Elena Martín-Ávila, María Martín-Martín, Sara Martín-Martínez, Anunciación Martínez-Arroyo, Carmen Martínez-Palomo, Cristina Martínez-Ruíz, Pilar Martínez-Zafra, Alicia Mateo-Madurga, Natalia Méndez-Junco, Antonia Minguito-Lobos, Paloma Molina-Gómez, Marina Moreno-Collado, Ana B. Moreno-Moreno, Cristina Olmos-Sancho, Remedios Peláez-Toré, Raquel Pérez-Barrios, Pérez-Barrios Pérez-García, Esmeralda Pulido-López, Ana B. Ramírez-Puerta, Luz del Rey-Moya, Araceli Rivera-Álvarez, Paz Rodrigo-Rodrigo, María N. Ruiz-Martín, AnaI Serna-Urnicia, Josefa Sidera-Jiménez, Encarnación Tornay-Muñoz, Laura Villanova-Cuadra, Isabel Villanueva-Alameda, Cristina Villanueva-Sanz, Emiliana Villares-Motino

**Affiliations:** 1 Research Unit, Primary Care Assistance Management, Madrid Health Service, Madrid, Spain; 2 Health Services Research On Chronic Patients Network (REDISSEC) and Research Network On Chronicity, Primary Health Care and Health Promotion (RICORS-RICAPPS), Instituto de Salud Carlos III, Madrid, Spain; 3 Gregorio Marañon Health Research Institute, Madrid Health Service, Madrid, Spain; 4 Doctoral Program in Epidemiology and Public Health (Interuniversity), Rey Juan Carlos University, Madrid, Spain; 5 Nursing Department, Faculty of Nursing, Physiotherapy and Podiatry, Complutense University of Madrid, Madrid, Spain; 6 Independent researcher, Preventive Medicine and Quality Management Service, Gregorio Marañón General University Hospital, Madrid, Spain; 7 Preventive Medicine and Public Health Area, Health Sciences Faculty, Rey Juan Carlos University, Alcorcón, Madrid, Spain; 8 Goya Healthcare Center, Primary Care Assistance Management, Madrid Health Service, Madrid, Spain; 9 Foundation for Biosanitary Research and Innovation in Primary Care of the Community of Madrid (FIIBAP), PEJD-2018-PRE / BMD-9460, Madrid, Spain; 10 Primary Care Assistance Management, Madrid Health Service, Madrid, Spain

**Keywords:** Quality of life, Gender roles, Caregivers, Informal care, Primary health care, Chronic disease, Disabled persons

## Abstract

**Background:**

Most care for people with chronic or disabling conditions living in the community is provided in the family context, and this care is traditionally provided by women. Providing informal care has a negative impact on caregivers’ quality of life, which adds to existing health inequalities associated with gender. The aim of this study was to analyze factors associated with the health-related quality of life of caregivers and to determine their differences in a gender-differentiated analysis.

**Methods:**

An observational, cross-sectional, multicenter study was conducted in primary healthcare. A total of 218 caregivers aged 65 years or older were included, all of whom assumed the primary responsibility for caring for people with disabling conditions for at least 6 months per year and agreed to participate in the CuidaCare study. The dependent variable was health-related quality of life, assessed with the EQ-5D. The explanatory variables tested were grouped into sociodemographic variables, subjective burden, caregiving role, social support and variables related to the dependent person. The associations between these variables and health-related quality of life were estimated by fitting robust linear regression models. Separate analyses were conducted for women and men.

**Results:**

A total of 72.8% of the sample were women, and 27.2% were men. The mean score on the EQ-5D for female caregivers was 0.64 (0.31); for male caregivers, it was 0.79 (0.23). There were differences by gender in the frequency of reported problems in the dimensions of pain/comfort and anxiety/depression. The variables that were associated with quality of life also differed. Having a positive depression screening was negatively associated with quality of life for both genders: -0.31 points (95% CI: -0.47; -0.15) for female caregivers and -0.48 points (95% CI: -0.92; -0.03) for male caregivers. Perceived burden was associated with quality of life in the adjusted model for women (-0.12 points; 95% CI: -0.19; -0.06), and domestic help was associated in the adjusted model for male caregivers (-0.12 points; 95% CI: -0.19; -0.05).

**Conclusions:**

Gender differences are present in informal caregiving. The impact of providing informal care is different for male and female caregivers, and so are the factors that affect their perceived quality of life. It could be useful it incorporates a gender perspective in the design of nursing support interventions for caregivers to individualize care and improve the quality of life of caregivers.

**Trial registration:**

NCT 01478295 [https://ClinicalTrials.gov]. 23/11/2011.

**Supplementary Information:**

The online version contains supplementary material available at 10.1186/s12912-022-00845-x.

## Introduction

By 2050, more than 2 billion of the world's population will be aged 60 years or over, and in Europe, the most rapidly aging region, the proportion will exceed 30% [1]. In Spain, people over 65 years of age will account for 25.2% of the population in 2033 [2]. This demographic change is leading to a higher prevalence of chronic diseases such as neurocognitive disorders, diabetes, cardiovascular and pulmonary diseases, cancer and osteoarticular problems [1]. In our region, ageing and multimorbidity account for increasing numbers of patients living with multiple complex chronic conditions. This are associated with different levels of dependency for basic activities of daily living that require a combination of self-care actions provided by informal caregivers and professional care at home [3].

Different models of care provision can be found in Europe according to the degree of government and institutional involvement, financial benefits and formal support to family caregivers [4]. In most countries, particularly in Mediterranean area, the majority of caregiving is provided by informal caregivers [5, 6] in family and domestic contexts [7, 8], and it is estimated that on average, approximately 61% of informal caregivers in Organization for Economic Co-operation and Development countries are women [7, 9]. However, social and cultural trends are changing the traditional patterns of care. Women's increased participation in the economic and social spheres, their growing incorporation into the labor market and the transformation of the family structure are generating a conflict between the demands of the economy, social transformation and the increase in both the need for and the complexity of informal care [10–12].

Research shows that long-term informal care has a negative impact on caregiver health and is associated with poor health-related quality of life and increases in perceived burden, stress and anxiety [8, 13–17]. This impact is different for female caregivers, who have more difficulty falling asleep, a worse perceived state of health and higher rates of anxiety, depression, pain and discomfort than male caregivers [11, 18]. These studies also show that factors such as the caregiver’s age, gender and marital status can increase or decrease the impact of informal caregiving on their health. The relationship to the dependent person, the person’s level of dependency, the intensity of care he or she requires and the formal support received also have an impact [19–21].

The SARS-CoV-2 disease 2019 pandemic has contributed additional challenges for family caregivers. Domiciliary confinement, quarantine, social isolation, difficulty in providing access to health services and the discontinuation of social support services have been the main factors that have decreased the functional ability of disabled people and have increased the burden and complexity of their daily care, negatively impacting the health and quality of life of caregivers [22–28].

A study performed in Serbia to explore the quality of life of informal caregivers showed a subjective decrease compared to the pre-pandemic experience, associated with an increased burden and complexity of care, concern for their own health and that of the person they are caring for [28]. These concerns are described in other studies, along with limited opportunities to maintain their personal well-being and the need to modify the caregiving approach by assuming new responsibilities and managing a new caregiving routine [25]. In general, caregivers report an increase in depressive symptoms, stress, anxiety and burden [22, 23, 26, 27, 29, 30].

Research that incorporates a gender-differentiated analysis shows that the factors that impact burden and quality of life are different and are related not just to the caregiving role but to existing gender inequalities [31–34]. In research conducted in Mediterranean countries, men who serve as primary family caregivers generally do so only when female care resources have been exhausted [35].

The WHO highlights the need to recognize, support and consolidate informal care as a fundamental element of the present and future health care of the European population [10]. Measuring health-related quality of life can help summarize the experience of caregivers and therefore characterize the burden of caregiving. Understanding the relationships between factors associated with health-related quality of life, including from a gender perspective, can be of great interest in planning interventions to improve health outcomes.

The objective of this study was to identify factors associated with the health-related quality of life of caregivers for community-dwelling people with chronic or disabling conditions, examining differences in a gender-specific analysis. Secondarily we aimed to determine if there were any differences in the five dimensions of quality of life between male and female caregivers.

## Methods

### Aim

To identify factors associated with the health-related quality of life of caregivers for community-dwelling people with chronic or disabling conditions, examining differences in a gender-specific analysis.

The secondary objective was to determine if there were any differences in the five dimensions of quality of life between male and female caregivers.

### Design and setting

This was an observational, cross-sectional, multicenter study based on data from the baseline visit of the CuidaCare study [36]. The CuidaCare study was a pragmatic, multicenter, two-armed, cluster-randomized controlled trial with a twelve-month follow-up conducted at twenty-two primary health care centers in five municipalities of the Madrid region. These centers served a total of 549,203 people. Eighty-nine nurses participated in the study. The STROBE checklist is available as supporting information (Additional file 1).

### Participants

Caregivers included were 65 years or older who assumed primary responsibility for caring for a person with a physical or mental disabilities (functional impairment, elderly, neurocognitive disorders, advanced chronic diseases or palliative care needs) for at least 6 months per year, were able to participate in the study and had given their written informed consent to participate in the CuidaCare study. We excluded caregivers who were already receiving therapeutic interventions to decrease their burden and those who were caring for nursing home residents or hospitalized people during the recruitment period. Patients have named nurses who are responsible for providing and coordinating their care. Nurses identified caregivers who met the study selection criteria, during routine nursing consultations at the primary care health center or at home visits. They offered caregivers to participate consecutively and requested their informed written consent to participate before healthcare centers randomization, between February and May 2014 [36].

### Sample and sampling procedure

The estimated sample size for the CuidaCare study was 142 caregivers. The sample assumptions and calculation are detailed in the protocol published in this journal [37].

### Data collection procedure and variables

All of the variables described below were collected in June 2014 via interviews conducted by nurses and were recorded in an electronic data collection notebook. The nurses were previously trained.

The dependent variable was health-related quality of life, measured with the EQ-5D-3L instrument. The EQ-5D questionnaire is a generic standardized instrument developed to describe and assess health-related quality of life. It consists of two parts: a visual analog scale (VAS) on which the respondent scores his or her health between two extremes, 0 and 100, which represent the worst and best imaginable state of health, respectively, and a descriptive system that comprises 5 dimensions (mobility, self-care, usual activities, pain/discomfort and anxiety/depression) and offers three response levels for each dimension: no problem, some problems and extreme problems/inability. A single weighted score (utility index) was also calculated. These utility values were obtained from the algorithm proposed for our country [38].

The explanatory (independent) variables tested were grouped into sociodemographic variables such as the caregiver’s age, marital status, relationship to the dependent person and educational level; subjective burden, including perceived anxiety (Goldberg Anxiety Inventory), perceived depression (Yesavage Geriatric Depression Scale) and perceived burden (Caregiver Strain Index); variables related to living and caregiving conditions, such as formal support and family function (family Apgar); and variables related to the care recipients, such as multimorbidity, performance of activities of daily living (Barthel scale) and cognitive skills (Pfeiffer test).

The gender of the caregiver (male or female) was used for a differentiated analysis.

### Data analysis

A descriptive analysis of the characteristics of the caregivers and the people with chronic or disabling conditions for whom they cared was performed using frequencies and percentages for qualitative variables and means and standard deviations (SD) or medians and interquartile ranges (IQR) for quantitative variables according to their distribution. The mean quality of life (VAS and utility index) and the prevalence of problems in any of the five dimensions was estimated. For a better understanding, the presence or absence of problems was dichotomized. Qualitative variables were compared using the Pearson chi-squared test, and normally distributed quantitative variables were compared using Student’s t-test. To examine the factors associated with health-related quality of life, linear regression models were fitted. Considering that the caregivers were included in the study through cluster sampling, robust estimators were obtained. Three models were fitted using the VAS score as the dependent variable, and three were fitted using the utility index score as the dependent variable. Considering possible gender-related differences, separate models were constructed for the total sample, for male caregivers and for female caregivers. All analyses were performed using STATA 14 software.

## Results

Of the 224 caregivers included in the study, 163 (72.8%) were women, and 61 (27.2%) were men. Table 1 shows the characteristics of caregivers and the differences between the men and women.

**Table 1:** Gender differences in sociodemographic characteristics, subjective burden, caregiving role and social support

	**Total (*n* = 224)**	**Female 163 (72.8%)**		**Male 61 (27.2%)**	***p***
	n (%)	n (%)		n (%)	
	mean (SD)	mean (SD)		mean (SD)	
	median (IQR)	median (IQR)		median (IQR)	
**Sociodemographic characteristics**					
Age	78.1 (6.9)	76.7 (6.7)		81.7 (6.4)	** < 0.001**
Marital status					
Married	192 (85.7%)	135 (82.8%)		57 (93.4%)	**0.043**
Single/Divorced/Widow	32 (14.3%)	28 (17.2%)		4 (6.6%)	
Familiar relationship					
Spousal	160 (71.4%)	109 (66.9%)		51 (83.6%)	**0.014**
Ancestor/Descendant/Other	64 (28.6%)	54 (33.1%)		10 (16.4%)	
Educational level					
Primary or less (≤ 12 years)	156 (69.6%)		118 (72.4%)	38 (62.3%)	0.143
Secondary or above (> 12 years)	68 (30.4%)		45 (27.6%)	23 (37.7%)	
Employment status					
Unpaid domestic work	78 (34.8%)		77 (47.2%)	1 (1.6%)	** < 0.001**
Employed/Unemployed/ Retired	146 (65.2%)		86 (52.8%)	60 (98.4%)	
**Subjective burden**					
Burden (Caregiver Strain Index), (yes)	102 (46.8%)		79 (50.0%)	23 (38.3%)	0.123
Anxiety (Goldberg), (yes)	118 (54.1%)		96 (60.8%)	22 (36.7%)	**0.001**
Depression (Yesavage) (yes)	28 (12.9%)		25(15.8%)	3 (5.0%)	**0.033**
**Caregiving role**					
*Living conditions*					
Living with care recipient only, (yes)	148 (66.1%)		104 (63.8%)	44 (72.1%)	0.241
Household size	2 (2–3)		2.7 (1.5)	2.3 (0.7)	0.158
*Caregiving conditions*					
Experience as a caregiver, (years)	6 (3–10)		9.2 (9.9)	8.2 (8.0)	0.730
Family function (Family Apgar), (dysfunctional)	55 (25.2%)		39 (24.7%)	16 (26.7%)	0.763
**Social support**					
*Dependency Law*					
Level recognized by government, (yes)	65 (29.0%)		50 (30.7%)	15 (24.6%)	0.372
Formal Support, (yes)	82 (36.6%)		56 (34.4%)	26 (42.6%)	0.253
Domestic helper, (yes)	72 (32.1%)		48 (29.5%)	24 (39.3%)	0.158
Domestic helper, (hours per week) (*n* = 72)	9.26 (28.4)		6.38 (20.3)	16.98 (5.4)	**0.013**

### Gender differences in sociodemographic characteristics

The mean age (± SD) was 78.1 years (± 6.9). The women were 4.9 years old younger than the men (95% CI: -6.87; -2.97). Of the male caregivers, 83.6% cared for their wives, while 33.1% of the female caregivers cared for other family members (*p* = 0.014). Seventy-eight of the caregivers in the sample (34.8%) had engaged in unpaid domestic work during their working lives, including 1 man (1.6%) and 77 women (47.2%) (*p* < 0.001).

### Gender differences in subjective burden

No relevant differences were found for the subjective burden measured using the Caregiver Strain Index. However, a significantly higher percentage of female caregivers than male caregivers perceived anxiety according to the Goldberg scale, 60.8% vs 36.7% (*p* = 0.001). A total of 28 caregivers in the sample presented positive depression test scores, and the percentage was 3 times higher in female caregivers, 15.8% vs. 5.0% (*p* = 0.033).

### Gender differences in caregiving role and social support

Regarding the conditions associated with the caregiving role and the social support received, the difference in the hours of domestic help received by male and female caregivers stands out: female caregivers received 10.6 fewer hours/week of support than the male caregivers (95% CI: -18.91; -2.29).

### Gender differences in health-related quality of life

Table 2 shows the perceived quality of life of the male and female caregivers, as measured with the EQ-5D-3L instrument. The mean perceived health score of the female caregivers, as measured with the EQ-5D VAS, was 54.9; this was 6.83 points lower than the males, whose mean perceived health score was 61.8 (95% CI: -13.25; -0.42).

Differences were also evident in the utility index. The female caregivers had a 0.148-point lower mean utility index than the males (95% CI: -0.235; -0.063), and their scores were 0.64 and 0.79 points, respectively.

**Table 2:** Gender differences in health-related quality of life

	**Total (*n* = 218)**	**Female 158 (72.5%)**	**Male 60 (27.5%)**	***p***
	n (%)	n (%)	n (%)	
	mean (SD)	mean (SD)	mean (SD)	
	median (IQR)	median (IQR)	median (IQR)	
**EQ-5D visual analogue scale and utility index value**				
VAS (mean (SD))	56.8 (21.6)	54.9 (21.9)	61.8 (20.2)	**0.036**
VAS (median, RIQ)	51 (48–70)	50 (45–70)	60 (50–80)	**0.032**
EQ-index (mean (SD))	0.68 (0.295)	0.64 (0.308)	0.79 (0.23)	** < 0.001**
EQ-index (median,RIQ)	0.82 (0.576–0.887)	0.75 (0.381–0.887)	0.89 (0.719–0.914)	** < 0.001**
**EQ-5D descriptive system**				
Mobility (some or extreme problems)	85 (39%)	65 (41.1%)	20 (33.3%)	0.291
Self-Care (some or extreme problems)	32 (14.7%)	27 (17.1%)	5 (8.3%)	0.103
Usual activities (some or extreme problems)	78 (35.8%)	61 (38.6%)	17 (28.3%)	0.158
Pain/ Disconfort (some or extreme problems)	171 (78.4%)	131 (82.9%)	40 (66.7%)	**0.009**
Anxiety/ Depression (some or extreme problems)	127 (58.3%)	102 (64.6%)	25 (41.7%)	**0.002**

**Fig. 1:**
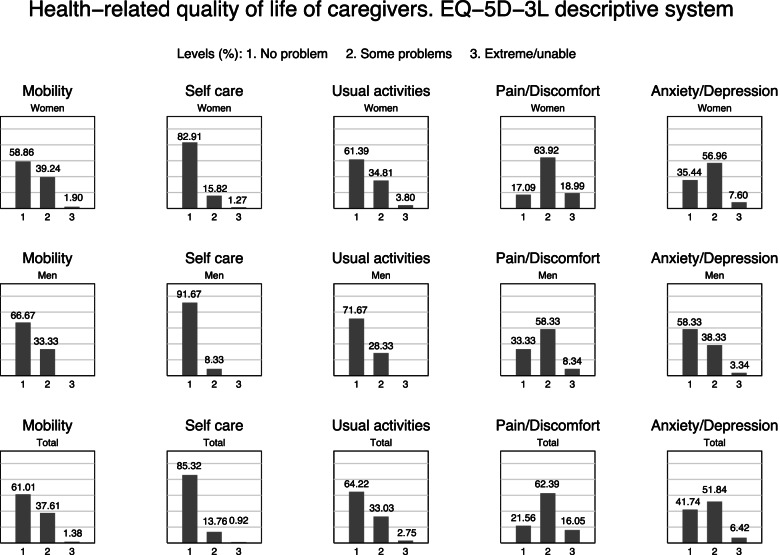
Health-related quality of life of caregivers, according to the EQ-5D-3L descriptive system

Among the five dimensions assessed by the EQ-5D, the dimensions of pain/discomfort and anxiety/depression included the highest percentage of caregivers with some or extreme problems (78.4% and 58.3% respectively), and there were significant between male and female caregivers. Figure 1 shows the differences in the dimensions of health-related quality of life.

### Gender differences in the care recipients’ sociodemographic characteristics and care needs

There were significant differences in the gender and marital status of the care recipients according to the caregiver's gender. Compared to female caregivers, who cared mostly for male dependents (71.6%), male caregivers only cared for other males in 6.6% of the cases (*p* < 0.001). The clinical and demographic characteristics of the dependents are shown in Additional file 2.

### Factors associated with health-related quality of life

Table 3 shows the factors associated with the caregivers' perceived quality of life. In the fitted global model using VAS scores, being a male caregiver was associated with a 3.91-point improvement (95% CI: -0.84; 8.67); however, this improvement was not statistically significant. Having positive anxiety screening results decreased the VAS score by -10.63 points (95% CI: -17.78; -3.48). Having positive depression screening results decreased the VAS score by -13.39 (95% CI: -23.56; -3.22), and having domestic help decreased it by -9.08 (95% CI: -15.33; -2.83). Having a family Apgar score in the functional range increased the health-related quality of life VAS score by 6.97 (95% CI: 1.79; 12.15). Caring for a severely or totally dependent person was also associated with a higher VAS score, with an improvement of 6.66 points (95% CI: 0.19; 13.13).

**Table 3:**
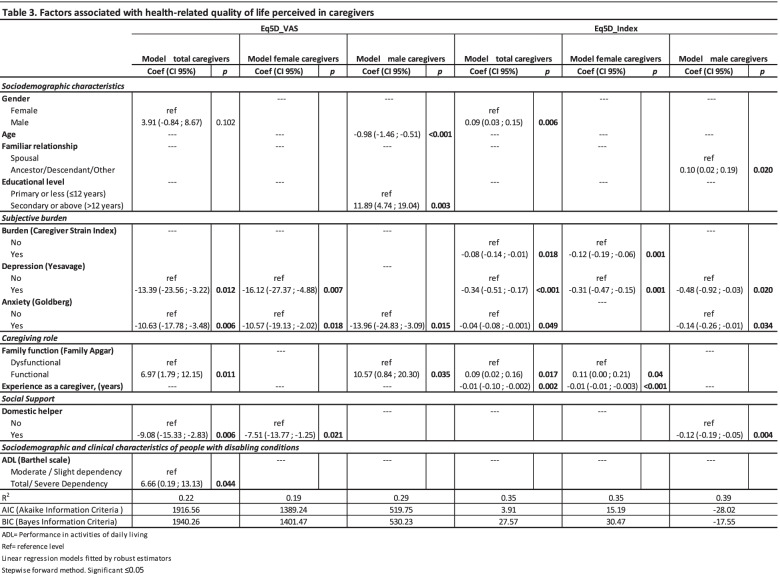
Factors associated with health-related quality of life perceived in caregivers

In the gender-differentiated analysis, differences were found in the factors that were related to perceived quality of life, as measured by the VAS score. In the fitted model for female caregivers, no variables were associated with an improvement in quality of life, while in the fitted model for male caregivers, having a secondary or higher education level improved the VAS score by 11.89 points (95% CI: 4.74; 19.04), and having a functional family Apgar score improved it by 10.57 points (95% CI: 0.84; 20.30). Factors that were negatively associated with the VAS score in the fitted female model included a positive depression screening result, which decreased the score by 16.12 points (95% CI: -27.37, -4.88). In the fitted male model, a positive anxiety screening result was associated with the greatest score decrease, reducing it by 13.96 points (95% CI: -24.83, -3.09).

In the fitted global model using the EQ-5D score, being a male caregiver was associated with a 0.09-point score increase (95% CI: 0.03; 0.15). All of the variables grouped under subjective burden (Caregiver Strain Index, positive depression screening result and positive anxiety screening result) were associated with a decrease in the perceived quality of life score. Detailed information on the six linear regression models is given in Table 3.

In the adjusted model for women, burden decreased the EQ-5D index score by 0.12 points (95% CI: -0.19; -0.06), and a positive depression screening decreased it by 0.31 points (95% CI: -0.47; -0.15). In addition, each year spent providing care decreased the index score by 0.01 points (95% CI: -0.01; -0.003). The perception of functional family relationships improved the women’s quality of life by 0.11 points (95% CI: 0.008; 0.21).

In the adjusted model for men, no association was found with any caregiving role variable. Positive depression and anxiety screening results decreased their quality of life scores by 0.48 points (95% CI: -0.92; -0.03) and 0.14 points (95% CI: -0.26; -0.01), respectively. Having domestic help also decreased perceived quality of life, by 0.12 points (95% CI: -0.19; -0.05). Caring for a non-spouse increased the index score by 0.10 points (95% CI: 0.02; 0.19).

## Discussion

### Main findings of the study and comparison with other studies

#### Gender and caregivers

The caregivers in the CuidaCare study were mostly women (72.8%), married (82.8%), without an education or with a primary education (72.4%), and who had always dedicated to domestic work (47.2%). This socioeconomic profile is consistent with the reports in the literature and similar to data from our region [15, 18, 19, 31, 32, 34, 39, 40]. Our study found a slightly higher proportion of male caregivers (27.2%) than has been reported in other studies. Soronellas and Comas-D'Argemir, in studies conducted in Spain, explained this increase in the involvement of men in family care as a consequence of the care crisis, the economic crisis and the reactivation of the protective role of kinship in a way that relegates gender roles to the background [41]. In our study, there were other factors that could explain this situation, such as the average age of the participants, which was 78.1 years; the high percentage of households in which the caregiver lived alone with the dependent person (66.1%); and the fact that 71.4% of the participants cared for their husband or wife. Abellán et al.’s study of care in Spain in 2018 found that with age, gender differences in family care decrease, and the proportion of male caregivers is increased among those over 65 years of age. In addition, with increasing age, the provision of partner care increases progressively, and in older households, the number of male and female caregivers is much more balanced [42].

### Gender differences in health-related quality of life

The results of our study suggest that there are also different factors associated with perceived quality of life as a function of the caregiver’s gender. As in other studies, the presence of alterations in mental health negatively influenced the quality of life scores of both genders [40, 43]. Only family support improved quality of life in both genders.

In female caregivers, lower quality of life scores were associated with the presence of burden and the number of years of caregiving, while in male caregivers, lower scores was associated with positive anxiety screening results and receiving domestic help. It would be interesting to study whether the presence of burden in women and receiving domestic help in men actually describe the same phenomenon (intensity of care).

In our study, no significant differences were found for subjective burden, although women showed higher levels of perceived anxiety and depression. These results are different from those found in the systematic review by Xiong et al. [32], which concluded that sex and gender differences in mental and physical health are limited, although they suggest differences in the burden of care. In this sense, others studies show that female caregivers register a higher burden [33].

This study [33] also suggests that women have partners with more caregiving needs and spent more hours per week on caregiving than male caregivers. However, we have not found significant differences in caregiving role, although men in our study benefited from more hours of domestic help per week.

The average quality of life reported by caregivers was lower than that of the general population aged 75 years or older in Spain for both genders [39] and lower than that reported by caregivers in the CUIDAR-SE study [18], which explored gender inequalities in the impact of informal care in a Spanish region. This difference from the CUIDAR-SE study could be related to the age of the participants, which was higher in the CuidaCare study, since age is negatively associated with health-related quality of life [19, 39, 43].

The female caregivers reported a lower quality of life than the male caregivers, although this phenomenon is also described in the general population [39, 43] and is similar to the findings of other studies of caregivers [18, 44].

### Gender differences in the dimensions of quality of life

The dimensions of quality of life that were most affected in caregivers of both genders were pain/discomfort and anxiety/depression, which are also the most affected dimensions in the general population [39, 43]. A higher proportion of women than men presented problems in at least one dimension of quality of life, a finding that is also consistent with related studies [18, 40].

### Strengths and limitations

The main limitation of this study is the age of the results. The delay in the publishing of the findings is due, on the one hand, to problems in the management of the funding granted for the study and, on the other, to the personal difficulties experienced by the research team. Afterwards, the acute outbreak and severity of the COVID pandemic in Spain made impossible for us to continue working on research, as we had to focus on patient care.

Despite this delay, the results found are still valid and are of interest to deepen caregiver support. In addition, the results of the effectiveness of nursing intervention to improve the quality of life of caregivers in the CuidaCare study have recently been published [36].

Caring for caregivers remains a priority for society and for the sustainability of healthcare systems, especially after the impact that the COVID-19 pandemic has had on vulnerable groups such as the elderly and caregivers [45–50]. Sedentary lifestyles and, as a consequence, frailty and functional impairment have increased in people over 85 years of age [47–49]. Caregivers have seen their burden of care increase and their well-being decrease [49]. In addition, the difficulty of accessing health services, due the use of new technologies, and social isolation, have increased anxiety and the feeling of abandonment of disabled people and their caregivers [45, 50].

The study has other limitations inherent to cross-sectional studies, namely, that causal associations cannot be established.

One of the strengths of the study is that the data were collected in the context of a pragmatic clinical trial, through clinical interviews and by the nurses who were assigned to these caregivers via their patient lists. In addition, these nurses had received training in the ad hoc collection of data. This pragmatic approach and the data collection method used favor the quality of the registry and approximate the reality of caregivers of community-dwelling people with chronic and disabling diseases.

The eligibility criterion that required that the subjects be participants in the CuidaCare clinical trial [36] could seem to be a limitation a priori, but in our environment, it is essential to focus on this age group, which corresponds to the age of informal caregivers in our clinical practice.

An additional strength is the use of health-related quality of life and its dimensions as the main outcome measure, since it was reported by the caregivers themselves as a measure of the impact of caregiving on their health.

In the CuidaCare clinical trial [36], patients were selected in the context of cluster sampling, which we accounted for in the analytical strategy by incorporating robust estimators in the regression models.

The last strength to highlight is the differentiation of the analysis by gender, which provides a necessary perspective given that there is a known inequity both in the assignment of roles within informal care and in the impact of informal caregiving on health.

### Implications of the study findings for clinical practice

In clinical practice, health-related quality of life and other outcomes reported by caregivers could be incorporated into nursing assessments as indicators of well-being and the impact of caregiver support interventions.

Knowledge of the gender inequalities that exist in informal care and the gender differences in both the impact of informal caregiving on health and the factors that influence the quality of life of male and female caregivers would facilitate the design of individualized nursing interventions to protect and improve the health of caregivers according to their gender.

In designing interventions, and taking into account the caregiver/dependent person dyad, it would be interesting to deepen the line of research of Bañez Tello [51] and incorporate into clinical practice the opinions and preferences of care recipients as they relate to the gender of the caregiver.

Health and social policies should consider care a social and shared responsibility and should not perpetuate existing gender differences in this role [52].

### Implications of the study findings for research

The results of this study can be the subject of future nursing research, where a gender perspective is taken into account during the design of studies. Integrating this approach into nursing research would allow us to contemplate a socio-sanitary vision of care for the caregivers.

## Conclusion

The results of the present study suggest that there is a negative impact of informal care on health-related quality of life, being greater for women than for men. Gender differences have also been found in the factors that are positively or negatively associated with quality of life. The dimensions most affected by informal care in both genders are pain/discomfort and anxiety/depression, but these problems occur more frequently in women.

Knowledge of the gender differences that exist in informal care and its impact on health and the factors that influence the quality of life and other outcomes reported by caregivers could be incorporated into nursing assessments as indicators of well-being. All this information should be considered in the design of individualized nursing interventions to promote and improve the health of caregivers according to their gender.

In designing of these interventions, it would be interesting to deepen the line of research propousal by Bañez Tello [42] and taking into account the caregiver/dependent person dyad to incorporate into clinical practice the opinions and preferences of care recipients as they relate to the gender of the caregiver.

## Supplementary Information

**Additional file 1.**

**Additional file 2.**

## Data Availability

The data that support the findings of this study are available from the Research Unit of the Madrid Primary Care Healthcare Management, but there are ethical and legal restrictions on sharing the data set because they contain sensitive clinical information about the caregivers and people with chronic and disabling conditions. The Ethics Committee approved this research without considering the option of data sharing. Therefore these data are not publicly available. The CuidaCare group may establish future collaborations with other groups based on these data, in which case, the main researcher [MRB] will be contacted. Each new project based on these data must be submitted in advance to the Ethics Committee for approval.

## Abbreviations

ADLs: Activities of daily living
IQR: Interquartile range
SD: Standard deviation
VAS: Visual analog scale
